# Case Report: Atezolizumab plus bevacizumab for combined hepatocellular-cholangiocarcinoma

**DOI:** 10.3389/fonc.2023.1234113

**Published:** 2023-07-21

**Authors:** Tomoyuki Satake, Taro Shibuki, Kazuo Watanabe, Mitsuhito Sasaki, Hiroshi Imaoka, Shuichi Mitsunaga, Motohiro Kojima, Masafumi Ikeda

**Affiliations:** ^1^ Department of Hepatobiliary and Pancreatic Oncology, National Cancer Center Hospital East, Kashiwa, Japan; ^2^ Division of Pathology, Exploratory Oncology Research and Clinical Trial Center, National Cancer Center Hospital East, Kashiwa, Japan

**Keywords:** combined hepatocellular-cholangiocarcinoma, atezolizumab, bevacizumab, immunotherapy, case series

## Abstract

Combined hepatocellular cholangiocarcinoma (cHCC-CCA) is a rare subtype of primary liver cancers. Therapeutic strategies for patients with cHCC-CCA are limited, and no standard systemic treatment has been established for unresectable cHCC-CCA. Here, we present six cases of cHCC-CCA treated with atezolizumab plus bevacizumab. We observed three partial responses and one stable disease as the best responses; two of these patients were still being treated with atezolizumab plus bevacizumab at the time of reporting (at least five months of treatment), whereas the remaining two patients were unable to continue treatment owing to adverse events. Atezolizumab plus bevacizumab may be an effective treatment for unresectable cHCC-CCA.

## 1 Introduction

Primary liver cancer, comprising chiefly hepatocellular carcinoma and cholangiocarcinoma, is the sixth most commonly diagnosed cancer and the third leading cause of cancer-related death worldwide, with an increasing incidence in most Western countries (1, 2). It is a heterogeneous group of tumors with distinct risk factors, clinical outcomes, and histological and molecular features. Combined hepatocellular-cholangiocarcinoma (cHCC-CCA) is a rare subtype comprising unequivocal and intimately mixed histopathological features of both hepatocellular carcinoma and cholangiocarcinoma within the same tumor (3). Its clinical outcome is considered poorer than that of hepatocellular carcinoma and equal to or worse than that of cholangiocarcinoma (4–7). Therapeutic strategies for patients with cHCC-CCA are limited; surgical resection is the only standard of care (8, 9). However, no standard systemic treatment has been established for patients with recurrent and/or advanced disease. Therefore, treatment regimens for hepatocellular carcinoma or cholangiocarcinoma have often been used as systemic therapies for cHCC-CCA. Recently, atezolizumab plus bevacizumab, a combination therapy of anti-programmed death ligand-1 (PD-L1) and anti-vascular endothelial growth factor (VEGF), was shown to be superior to sorafenib in the improvement both overall survival (OS) and progression-free survival (PFS) among patients with advanced hepatocellular carcinoma (10). However, few reports on the efficacy and safety of atezolizumab plus bevacizumab among patients with cHCC-CCA have been made. Herein, we report a series of six cases of unresectable cHCC-CCAs treated with atezolizumab plus bevacizumab. This study was approved by the Ethics Committee of the National Cancer Center in Japan (Approval No. 2021-477). Approval for review of the hospital records was obtained from the Institutional Review Board of the National Cancer Center, and informed consent was obtained from all patients.

## 2 Case descriptions

Characteristics of all the cases are summarized in **Table 1**.

**Table 1:** Characteristics of patients with combined hepatocellular-cholangiocarcinoma treated with atezolizumab plus bevacizumab.

Patient	Case 1	Case 2	Case 3	Case 4	Case 5	Case 6
Age, sex	62, Male	73, Female	76, Male	76, Male	74, Male	74, Male
Underlying liver disease	Chronic hepatitis	–	–	–	Chronic hepatitis	Fatty liver
Etiology of chronic liver disease	Hepatitis C virus	–	–	–	Hepatitis C virus	–
Child-Pugh classification (score)	B (8)	A (5)	A (5)	A (6)	A (5)	A (5)
UICC stage	T2N0M0 Stage II	T2N0M0 Stage II	T2N0M1 Stage IV	T3N1M0 Stage IIIB	T2N0M0 Stage II	T2N0M0 Stage II
BCLC stage	C	B	C	C	C	B
AFP (ng/mL) at baseline	27443	1.3	221.3	13.6	732	31.5
PIVKA-II (mAU/mL) at baseline	7366	39	21	72	82	48
CEA (ng/mL) at baseline	7.6	NE	3.7	3.2	6.7	65.8
CA19-9 (U/mL) at baseline	433.1	NE	42.9	99.3	33.3	28
Treatment regimen (1st line)	Atezolizumab plus bevacizumab	Atezolizumab plus bevacizumab	Atezolizumab plus bevacizumab	Atezolizumab plus bevacizumab	Gemcitabine and cisplatin plus S-1	Gemcitabine and cisplatin plus S-1
Treatment regimen (2nd line)	–	Gemcitabine and cisplatin plus S-1	Lenvatinib	–	Atezolizumab plus bevacizumab	Atezolizumab plus bevacizumab
Time on atezolizumab plus bevacizumab	1 y 3 m (ongoing)	4 m	2 m	6 m (ongoing)	5 m	1 m
Best tumor response	PR	PR	PD	SD	PR	PD
Adverse events with atezolizumab plus bevacizumab	Hypertension gr 3 Eruption gr 2 Skin ulcer gr 1 AST/ALT increase gr 2	Interstitial lung disease gr 1 Epistaxis gr 1	Eruption gr 1	Proteinuria gr 2	Cerebral infarction gr 3 Hypertension gr 2 Proteinuria gr 2 Eruption gr 1	Myocarditis gr 4

Reference values: AFP <10.0 ng/ml; PIVKA-II <39.0 mAU/mL; CEA <5.0 ng/mL; CA19-9 <37.0 U/mL.

UICC, Union for International Cancer Control; BCLC, Barcelona Clinic Liver Cancer; AFP, alpha-fetoprotein; PIVKA-II, protein induced by vitamin K absence or antagonist-II; CEA, carcinoembryonic antigen; CA19-9, carbohydrate antigen 19-9; AST, aspartate aminotransferase; ALT, alanine aminotransferase; y, years; m, months; PR, partial response; PD, progressive disease; SD, stable disease; NE, not evaluated; gr, grade. "-" meant no applicable item.

### 2.1 Case 1

**Figure 1:**
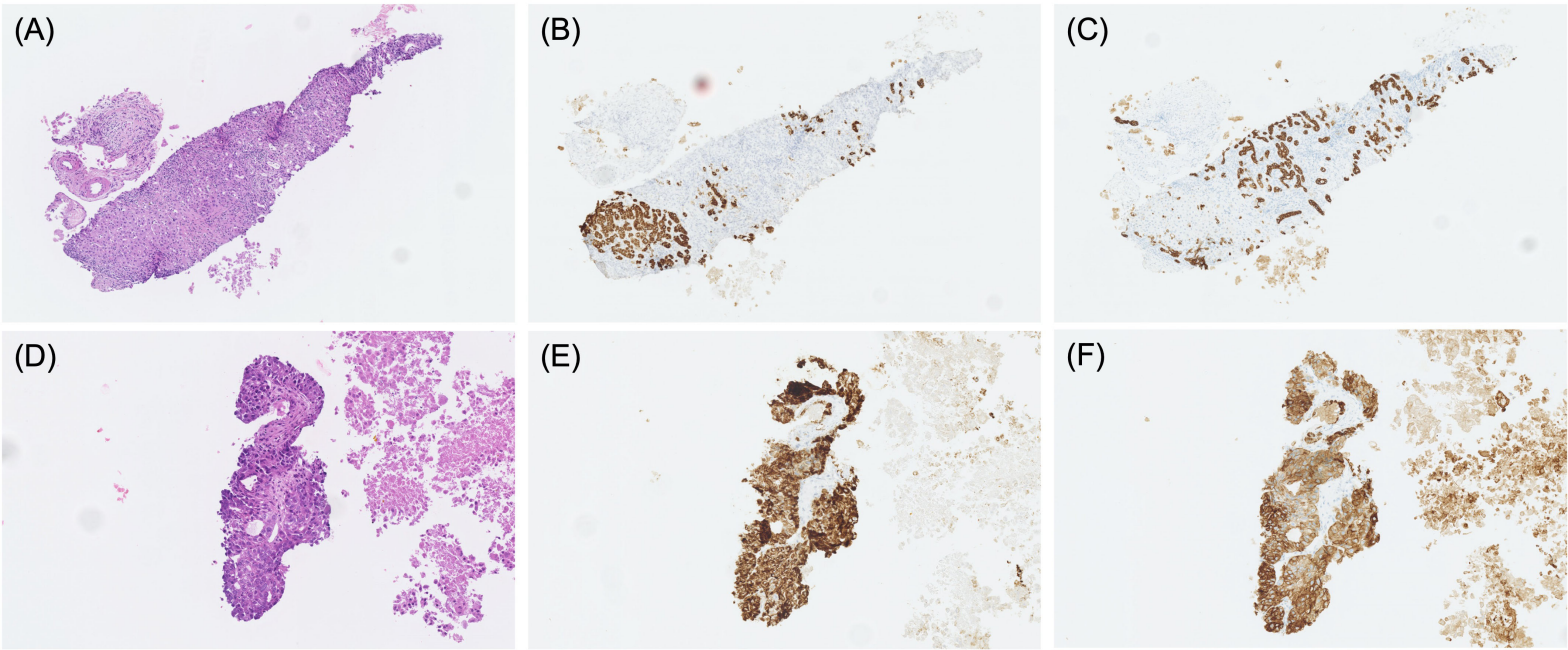
Pathological findings of specimens obtained by percutaneous liver biopsy in cases 1 **(A–C)** and case 4 **(D–F)**. HE staining **(A, D)**, hepatocyte staining **(B, E)**, and CK19 staining **(C, F)**.

**Figure 2:**
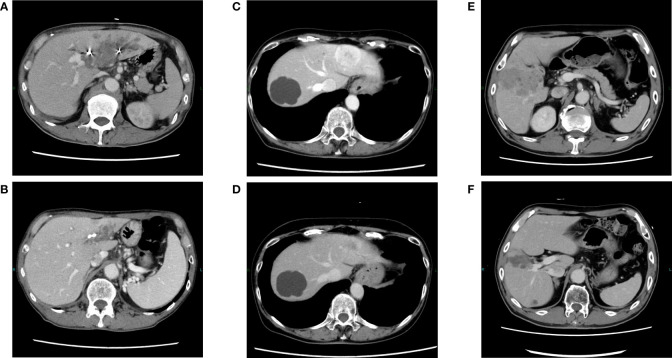
Changes in contrast-enhanced CT findings in responders during treatment. Pretreatment with atezolizumab plus bevacizumab in cases 1 **(A)**, 2 **(C)**, and 5 **(E)**. After treatment with atezolizumab plus bevacizumab in cases 1 **(B)**, 2 **(D)**, and 5 **(F)**, in which a partial response was observed.

A 62-year-old man presented to our hospital with a liver tumor due to cholangitis via computed tomography (CT). He was diagnosed with liver cancer containing an adenocarcinoma component via a bile duct biopsy obtained during endoscopic biliary drainage. An additional liver biopsy was performed, and immunohistochemistry revealed atypical cells with bidirectional hepatocellular differentiation, with the expression of hepatocytes, and ductal differentiation, with CK19 expression, leading to a diagnosis of cHCC-CCA (**Figure 1**). The liver tumor was unresectable because it had extensively invaded the main portal vein. At the start of treatment, the patient had Child-Pugh class B liver function, but his cHCC-CCA was Barcelona Clinic Liver Cancer stage C. We decided that systemic therapy was preferable to transcatheter arterial chemoembolization or transcatheter arterial infusion, and first-line therapy with atezolizumab plus bevacizumab was initiated. Regarding the therapeutic effect, the levels of tumor markers, such as alpha-fetoprotein (AFP) and protein induced by vitamin K absence or antagonist-II (PIVKA-II), had decreased one month after the start of treatment, and a partial response (PR; based on the Response Evaluation Criteria in Solid Tumors version 1.1) was observed four months after the start of treatment (**Figures 2A, B**). Regarding adverse events, grade 3 hypertension was observed immediately after the start of treatment but was well controlled with the addition of antihypertensive drugs. In addition, eruptions appeared in the second cycle, but atezolizumab plus bevacizumab was continued while treating the eruption with topical steroids. However, the eruption did not improve sufficiently, and oral steroid treatment was required from the fourth cycle onward. At the same time, skin ulcers and acneiform eruptions, thought to be caused by bevacizumab, were observed. As mild liver dysfunction was also observed, atezolizumab plus bevacizumab was discontinued. Oral steroids and the addition of minocycline reduced the acneiform eruptions, enabling gradual tapering of these oral medications. The eruption became controllable with topical drugs alone, and after two weeks without it, atezolizumab plus bevacizumab was resumed. The tumor continued to shrink and the patient was still receiving atezolizumab plus bevacizumab one year and three months after the start of treatment.

### 2.2 Case 2

A 73-year-old woman was referred to our hospital because of liver tumors identified upon abdominal ultrasonography during a physical examination. Multiple hypervascular liver tumors occurred in both lobes of the liver. At the time, these tumors were judged to be unresectable hepatocellular carcinoma based on radiographic imaging features. First-line therapy with atezolizumab plus bevacizumab was initiated. Regarding the treatment effect, a PR was observed upon CT two months after the start of treatment (**Figures 2C, D**). As for adverse events, pneumonitis was discovered upon CT four months after the start of treatment. As the pneumonitis might have been drug-induced, the atezolizumab plus bevacizumab was discontinued and bronchoscopy was performed. During the bronchoscopy, a biopsy specimen was obtained, which revealed no tumor content. The patient recovered from the pneumonitis one month after treatment discontinuation. A diagnosis of atezolizumab-induced interstitial lung disease was made, and a liver biopsy was performed to examine the change from that treatment to the next. Immunohistochemistry confirmed adenocarcinoma components, including ductal differentiation, with CK7 expression, and hepatocellular differentiation, with the expression of hepatocytes, leading to a pathological diagnosis of cHCC-CCA. Considering the risk of recurrence of interstitial lung disease when resuming treatment with atezolizumab, we decided to switch to second-line therapy with gemcitabine plus cisplatin and S-1 (GCS). The disease was well controlled after initiation of GCS therapy, and gradual tumor shrinkage was observed. The tumor continued to shrink, and the patient was still alive two years and three months after starting atezolizumab plus bevacizumab treatment, receiving GCS therapy.

### 2.3 Case 3

A 76-year-old man was diagnosed with prostate cancer upon discovery of an elevated prostate-specific antigen concentration, and a CT scan revealed a liver tumor. A liver biopsy was performed, and immunohistochemistry revealed atypical cells with bidirectional hepatocellular differentiation, with the expression of hepatocytes, and ductal differentiation, with CK19 expression, leading to a diagnosis of cHCC-CCA. The liver tumor was unresectable, with portal vein invasion and bone metastasis, and first-line therapy with atezolizumab plus bevacizumab was initiated. Initially, the only adverse events were grade 1 skin eruptions, and treatment was continued. However, two months after the start of treatment, the patient developed progressive disease (PD), and atezolizumab plus bevacizumab was discontinued. Lenvatinib was started as second-line therapy, but PD was observed 2 months later, and lenvatinib was discontinued. In addition, thoracic aortic dissection was confirmed at that time, following which systemic treatment for cHCC-CCA was discontinued. Finally, best supportive care was provided in another hospital.

### 2.4 Case 4

A 76-year-old man was diagnosed with bladder cancer because of hematuria, and a CT scan at the time revealed liver tumors. Endoscopic ultrasound-guided tissue acquisition was performed, and immunohistochemistry revealed atypical cells with bidirectional hepatocellular differentiation, with the expression of hepatocytes, and ductal differentiation, with CK7/CK19 expression, leading to a diagnosis of cHCC-CCA (**Figure 1**). The patient had multiple liver tumors that were unresectable owing to multiple lymph node metastases, and first-line therapy with atezolizumab plus bevacizumab was initiated. The only adverse event was grade 2 proteinuria, and the treatment was continued. Two months after treatment initiation, the levels of AFP and PIVKA-II had decreased to normal levels, and stable disease (SD) with minor shrinkage was observed. At six months since the start of treatment, atezolizumab plus bevacizumab treatment was ongoing.

### 2.5 Case 5

A 74-year-old man with a history of proton therapy for hepatocellular carcinoma was regularly followed up. Four years and seven months after the proton therapy, multiple liver tumors were observed. Liver biopsy revealed poorly differentiated atypical cells that required differentiation between hepatocellular carcinoma and intrahepatic cholangiocarcinoma, and immunohistochemistry was positive for CK7/CK19; therefore, the patient was diagnosed with carcinomas mainly composed of adenocarcinoma. As multiple tumors were detected in each lobe and were unresectable, first-line treatment with GCS therapy was initiated. Two months after the start of treatment, PD was observed, and GCS was discontinued. Another liver biopsy revealed that the tumor was mainly composed of atypical cells with hepatocellular differentiation, with the expression of hepatocytes. Combined with the previous biopsy results, the tumor was diagnosed as cHCC-CCA. Atezolizumab plus bevacizumab was initiated as the second-line therapy. Two months later, PR was observed, and the levels of AFP and PIVKA-II had also decreased (**Figures 2E, F**). Subsequently, the treatment was continued while shrinkage was maintained; however, cerebral infarction developed before the seventh cycle of treatment. Owing to the aftereffects of cerebral infarction, it was difficult to continue systemic treatment for cHCC-CCA thereafter, and best supportive care was provided in another hospital.

### 2.6 Case 6

A 74-year-old man had previously been treated for follicular lymphoma and was followed-up regularly. Four years after partial remission of the lymphoma, multiple liver tumors were observed. Liver biopsy revealed that the main component was adenocarcinoma, including ductal differentiation with a slight mixture of atypical cells with hepatocellular differentiation, with the expression of hepatocytes, leading to a diagnosis of cHCC-CCA. Multiple unresectable tumors were discovered in both lobes of the liver, and first-line treatment with GCS therapy was initiated. Two months after the start of treatment, PD was observed, and GCS was discontinued. When liver biopsy was performed again, immunohistochemistry results were positive for hepatocytes, with a mixture of atypical cells exhibiting hepatocyte differentiation, consistent with the diagnosis of cHCC-CCA. Atezolizumab plus bevacizumab was initiated as second-line therapy. Three weeks after the start of administration, malaise and anorexia appeared, and liver and cardiac enzyme concentrations also increased. Electrocardiography revealed ST-segment changes, and coronary angiography revealed no evidence of ischemic heart disease. Myocardial biopsy revealed inflammatory cell infiltration, including of lymphocytes, leading to a diagnosis of immune-related acute lymphocytic myocarditis. Administration of 1000 mg of methylprednisolone was initiated for 3 days, and prednisolone (1 mg/kg) was continued thereafter. Although no marked deterioration in cardiac function was observed, the patient’s malaise and anorexia gradually worsened, even after the start of steroid treatment. CT revealed rapid tumor progression. The patient developed ascites, rapidly progressed to liver failure, and died two weeks after the onset of myocarditis.

## 3 Discussion

Combined hepatocellular-cholangiocarcinoma is a rare type of primary liver cancer; its reported incidence among primary liver cancers varies from 0.4% to 14.2% (11–15). This large incidence range is probably related to the evolving definition of cHCC-CCA over time in highly heterogeneous studies. Based on the National Cancer Registry, we previously reported that 0.53% of all primary liver cancers in Japan are cHCC-CCA (11). However, its true incidence is likely underestimated, as many patients have not undergone surgical resection. In such cases, cHCC-CCA may be misdiagnosed as hepatocellular carcinoma or cholangiocarcinoma by clinical diagnosis using imaging studies (12). Combined hepatocellular-cholangiocarcinoma is more common among men and individuals with cirrhosis or chronic liver disease caused by hepatitis B or C viral infections, and these features are more similar to hepatocellular carcinoma than to cholangiocarcinoma; therefore, certain cHCC-CCAs may be diagnosed as hepatocellular carcinoma if no biopsy is performed (16–19). In our cases, all patients underwent liver biopsy, and pathological evaluation, including immunostaining, led to a diagnosis of cHCC-CCA. In case 2, the patient was diagnosed with hepatocellular carcinoma based solely on radiographic imaging features without biopsy, but cHCC-CCA could be diagnosed via subsequent liver biopsy to confirm the pathology. In cases 5 and 6, cHCC-CCA was diagnosed from the beginning by liver biopsy, but the degree of mixture of hepatocellular carcinoma and cholangiocarcinoma components was different from that of the first biopsy in the pathological results of the second liver biopsy. These cases illustrate the complexity of accurately diagnosing cHCC-CCA using radiographic imaging or biopsy specimens, due to its heterogeneity.

Owing to its rarity, very little evidence and no guidelines are available for systemic treatment for cHCC-CCA, which is an unmet need. In many cases, cHCC-CCA does not have so-called “actionable” genetic alterations; thus, molecularly targeted therapy is considered only in select cases (20, 21). On the other hand, in recent years, development of treatments for hepatocellular carcinoma and cholangiocarcinoma using immune checkpoint inhibitors has progressed. In hepatocellular carcinoma, a randomized controlled trial revealed that atezolizumab plus bevacizumab significantly improved overall survival to a greater extent than sorafenib; accordingly, it is now widely used as the standard of care (10). In cholangiocarcinoma, a randomized controlled trial revealed that durvalumab plus gemcitabine and cisplatin significantly improved overall survival to a greater extent than gemcitabine and cisplatin, also becoming the standard of care (22). In addition, approximately 60% of cHCC-CCA cases belong to a particular subclass characterized by substantial immune infiltration, with activation of various pathways related to both innate and adaptive immunity and enrichment of several gene signatures associated with the response to immunotherapy in patients with hepatocellular carcinoma (23). Considering these results, we hypothesized that atezolizumab plus bevacizumab would yield therapeutic effects in cHCC-CCA; however, except for two case reports, no literature on immunotherapy for cHCC-CCA is available (24, 25).

Herein, we report the largest case series of unresectable cHCC-CCAs treated with atezolizumab plus bevacizumab. In this series, the best responses were PRs (three cases) and SD (one case), with only two patients exhibiting no improvement with atezolizumab plus bevacizumab. Positive responses to this treatment were observed when used as either first- or second-line therapy. These outcomes seem better than those demonstrated in previous reports of cHCC-CCA treatment (26–29). In a French multicenter retrospective study of 30 patients treated with gemcitabine combined with cisplatin or oxaliplatin, a PR was observed in 28.6% of patients, and the median PFS and OS were 9.0 and 16.2 months, respectively (26). In an American single-center retrospective analysis of 68 patients receiving systemic therapies, PRs to gemcitabine plus platinum-containing chemotherapy, gemcitabine plus fluorouracil, and sorafenib were observed in 24.3%, 15.4%, and 0% of patients, respectively (27). The median OS for these treatments was 11.5, 11.7, and 9.6 months, respectively and the median PFS was 8.0, 6.6, and 4.8 months, respectively. In a Japanese multicenter retrospective study of 36 patients, a PR or better was observed in 5.6% of the patients (28). The median OS in patients treated with gemcitabine plus cisplatin, fluorouracil plus cisplatin, and sorafenib was 10.2, 11.9, and 3.5 months, respectively and the median PFS was 3.0, 3.8, and 1.6 months, respectively. Multivariate analysis revealed that the OS of patients treated with sorafenib was inferior to that of those treated with platinum-containing therapy. In a recent single-center retrospective analysis from Korea, including 99 patients treated with sorafenib or cytotoxic chemotherapy, such as gemcitabine plus cisplatin and fluorouracil plus cisplatin, a PR or better was observed in 9.7% and 21.6% of patients, respectively, when used as first-line therapy (29). The median OS was 10.7 and 10.6 months, respectively and the median PFS was 4.2 and 2.9 months, respectively. When cytotoxic chemotherapy was divided into platinum-containing and non-platinum-containing chemotherapy, the response rate did not differ significantly. Taken together, all the available evidence suggests that platinum-containing chemotherapy is more effective than sorafenib as a treatment for unresectable cHCC-CCA. However, although our case series is limited by the small number of cases, our results indicate that atezolizumab plus bevacizumab may be even more effective than existing treatments for unresectable cHCC-CCA. As immunotherapy is effective against cHCC-CCA, it would be interesting to determine whether durvalumab plus gemcitabine and cisplatin, which is useful as a cholangiocarcinoma treatment, also has a therapeutic effect against cHCC-CCA. Further prospective studies are warranted to confirm these results in the absence of prospective trials of these agents.

Three patients in our case series had to discontinue treatment owing to adverse events. Interstitial lung disease and myocarditis were considered immune-related adverse events, and the possibility that the one case of cerebral infarction was also triggered by atezolizumab plus bevacizumab cannot be ruled out. All of these are known but rare adverse events of such treatment. Either multiple rare adverse events were observed by chance in our case series, or immune-related adverse events due to atezolizumab plus bevacizumab are more likely to occur in patients with cHCC-CCA than in those with hepatocellular carcinoma. Hence, further evaluation is required not only for the efficacy of this regimen but also for its safety.

## 4 Conclusion

Here, we presented a case series of patients with cHCC-CCA treated with atezolizumab plus bevacizumab. Our data showed promising effects of this treatment. This may be considered a reference case series for treatment selection for patients with this malignant disease, as no effective, evidence-based treatment is currently available. Further prospective investigations or studies with large numbers of cases are warranted to investigate the efficacy and safety of atezolizumab plus bevacizumab for unresectable cHCC-CCA.

## Data availability statement

The raw data supporting the conclusions of this article will be made available by the authors, without undue reservation.

## Ethics statement

Written informed consent was obtained from the participant/patient(s) for the publication of this case report.

## Author contributions

All authors were involved in the preparation of this manuscript. TSa examined patients, collected the data, and prepared the original draft. TSh MS, and HI examined patients, collected the data, and reviewed and edited the manuscript. KW, SM, MK, and MI reviewed and edited the manuscript. All authors contributed to the article and approved the submitted version.
